# Remote effects of a 7-week combined stretching and foam rolling training intervention of the plantar foot sole on the function and structure of the triceps surae

**DOI:** 10.1007/s00421-023-05185-5

**Published:** 2023-03-27

**Authors:** Andreas Konrad, Marina Maren Reiner, Anna Gabriel, Konstantin Warneke, Masatoshi Nakamura, Markus Tilp

**Affiliations:** 1 Institute of Human Movement Science, Sport and Health, University of Graz, Mozartgasse 14, 8010 Graz, Austria; 2 Professorship for Conservative and Rehabilitative Orthopedics, Technical University of Munich, Munich, Germany; 3 Institute for Exercise, Sport and Health, Leuphana University, Lüneburg, Germany; 4 Faculty of Rehabilitation Sciences, Nishi Kyushu University, Ozaki, Kanzaki, Saga Japan

**Keywords:** Non-local effects, Remote effects, Superficial back line, Stretching, Foam rolling

## Abstract

It is known that a single bout of foam rolling (FR) or stretching can induce changes in range of motion (ROM) and performance in non-directly adjoining areas of the dorsal chain (i.e., remote effects). However, to date, it is not known if such effects exist following long-term interventions. Thus, the purpose of this study was to investigate the remote effects of a 7-week combined stretching and FR training intervention of the plantar foot sole. Thirty-eight recreational athletes were randomly assigned to either an intervention (*n* = 20) or control (*n* = 18) group. The intervention group performed stretching and FR exercises of the plantar foot sole for 7 weeks. Before and after the intervention, the dorsiflexion ankle ROM, passive resistive torque at maximum angle (PRT_max_) and at a fixed angle, as well as maximum voluntary isometric contraction (MVIC) torque, were measured with a dynamometer. Gastrocnemius medialis and lateralis stiffness was assessed with shear wave elastography. The results showed no interaction effect for any of the parameters. There was a time effect indicating an increase in MVIC and PRT_max_, which was more pronounced in the intervention group (+ 7.4 (95% CI 2.5–12.4),  + 4.5 (95% CI − 0.2–9.2)) than the control group (+ 3.6 (95% CI − 1.4–8.6),  + 4.0 (95% CI − 2.2 to 10.2)). The results indicate no or minor remote effects of combined stretching and FR of the foot sole in the ankle joint. Potential non-significant changes in ROM were accompanied with an increase in stretch tolerance, but not with changes in muscle structure.

## Introduction

Stretching and foam rolling (FR) training is frequently performed in rehabilitation settings, and also in sports practice. Stretching training with its various methods (e.g., static stretching) for several weeks can induce chronic changes in local range of motion (ROM) (Medeiros and Martini 2018). It has been suggested that high-intensity and/or high-volume stretching must be applied to induce chronic changes in muscle–tendon unit properties, such as a decrease in stiffness (Freitas et al. 2018; Nakamura et al. 2021a, b). Otherwise, changes in the perception to stretch or stretch tolerance rather than structural changes are considered to be the main mechanisms responsible for the increase in ROM following stretching training over several weeks (Konrad and Tilp 2014; Freitas et al. 2018). In addition, it has been shown that high-volume stretching (> 30 min stretching per week) for several weeks can induce an increase in muscle strength (Yahata et al. 2021; Warneke et al. 2022a) as well as induce muscle hypertrophy (Warneke et al. 2022a, b, 2023). With regard to FR training, a recent meta-analysis showed that FR can increase quadriceps and hamstrings (but not triceps surae) ROM in the long term, if performed for more than 4 weeks (Konrad et al. 2022b). It is believed that such an increase in ROM after an FR intervention can be mainly attributed to increased stretch tolerance by, for example, tolerating higher passive torque values at the end ROM (Kasahara et al. 2022). With regard to performance parameters (e.g., maximum voluntary contractions, vertical jump height), another meta-analysis reported no changes following FR training interventions (ES = 0.294; *p* = 0.281) (Konrad et al. 2022a).

Although, in recent years, the evidence on the long-term local effects of stretching and FR training on various parameters, such as ROM, has grown, to date, there is no clear consensus on the long-term remote effects. Remote effects can be defined as non-local effects which might occur along myofascial chains linked via connective tissue. One of these chains, the dorsal chain, is often referred to as the superficial back line. The superficial back line extends from the plantar fascia over the Achilles tendon, the gastrocnemii muscles, and the hamstring muscles, through the back to the head (Stecco et al. 2019; Wilke et al. 2016). Various studies on acute effects have demonstrated that a single FR treatment on the plantar foot sole causes an increase in ROM of the hamstring muscles and the lower back (Grieve et al. 2015; Patel et al. 2016; Do et al. 2018). Moreover, a single treatment of the plantar foot sole through a combination of massage, FR, and stretching exercises resulted in an acute decrease in the performance of the dorsal chain (Gabriel et al. 2021). According to these studies, it can be assumed that muscle function can be acutely adjusted along the superficial back line. However, to date, it is not clear if this is the case for long-term training interventions.

Therefore, the purpose of this study was to investigate the remote effects of a 7-week combined stretching and FR training intervention of the plantar foot sole three times a week for 15 min per session on ankle ROM and strength and stiffness of the triceps surae muscles. We hypothesized that the 7-week intervention would increase dorsiflexion ankle ROM, but would not have any impact on plantar flexor strength. We further assumed that the change in ankle ROM would be associated with increased stretch tolerance (i.e., an increase in passive torque at maximum dorsiflexion ankle ROM), but without a decrease in stiffness of the triceps surae muscles.

## Materials and methods

### Participants

In a previous study (Konrad and Tilp 2014), a 6-week static stretching training program of the triceps surae muscles showed a large effect size (Cohen’s d = 1.1) for the increased ankle ROM. Hence, with the power to detect a large effect size, we calculated a minimum sample size of 15 participants for each group for this study (difference between two dependent means, effect size = 0.8, α = 0.05, 1 − β = 0.8) using G*Power software (G*Power version 3.1., Heinrich-Heine-University Düsseldorf, Germany) (Faul et al. 2009). Consequently, taking into account possible drop-outs, 38 healthy recreational athletes (female: *n* = 15, age: 28.4 ± 4.4 years, weight: 62.9 ± 7.4 kg, height: 167.9 ± 4.9 cm; male: n = 23, age: 26.4 ± 5.3 years, weight: 81.9 ± 7.2 kg, height: 183.3 ± 6.7 cm) volunteered in this study. All participants were free of any injury of the lower extremities and participated in a familiarization session to get used to the testing procedure. Although participants were informed about the testing procedure they were not informed about the hypotheses of this study. Possible risks and benefits were explained to the participants before they signed a written informed consent form. The ethical commission of the University of Graz consented to the ethical approval (approval code GZ. 39/68/63 ex 2020/2021), which conformed to the Declaration of Helsinki.

### Procedure

**Fig. 1:**
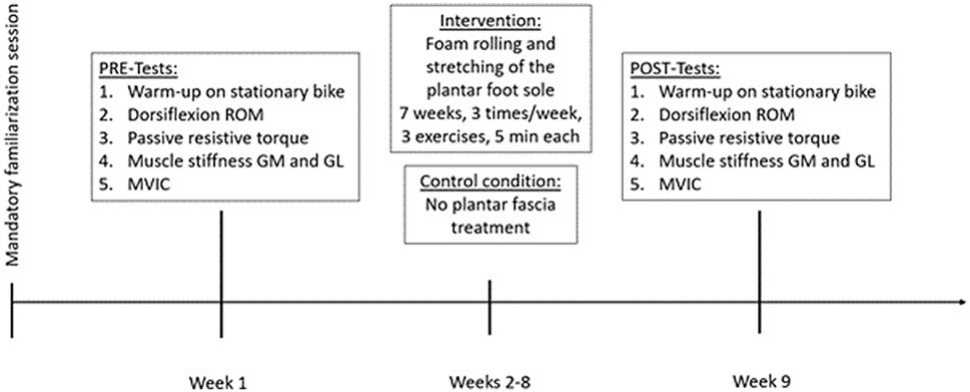
Schematic representation of the project procedure. Note: range of motion (ROM), gastrocnemius medialis (GM), gastrocnemius lateralis (GL), maximum voluntary isometric contraction (MVIC)

Each participant visited the laboratory three times—for a familiarization session, a pre-session before the 7-week intervention, and a post-session at the end of the intervention period. In the pre-session, the participants were randomly assigned to either the intervention or the control group by picking a hidden card (intervention group *n* = 20, control group *n* = 18). Males and females had separate cards to guarantee an equal distribution of the two sexes in both groups. Each appointment started with a 5-min warm-up on a stationary bike (Monark, Ergomedic 874 E, Sweden) at 60 rev.min-1 and 60 W. The measurements were performed on the dominant leg (i.e., the leg used for kicking a ball), and the following parameters were measured: ankle dorsiflexion ROM, passive resistive torque (PRT) in the ankle joint, maximum voluntary isometric contraction (MVIC) peak torque of the plantar flexor muscles, and muscle stiffness of the two gastrocnemius muscles (medialis (GM) and lateralis (GL)), assessed with shear wave elastography (SWE). Muscle activation level was measured via surface electromyography (sEMG) during the passive tests (ROM, PRT, SWE) to confirm that the participants were completely relaxed during the measurements. The order of the measurements is shown in Fig. 1, which was chosen to avoid any interfering effects between tests.

### Dorsiflexion range of motion (ROM)

For the dorsiflexion ROM testing, the participant was positioned prone on a bench with the ankle fixed on a dynamometer (Con Trex MJ, CMV AG, Dübendorf, Switzerland). The ankle joint axis of the dominant leg was aligned with the rotation axis of the dynamometer using a custom-made laser device, and the foot was fixed to the footplate with straps. The exact position was noted during the familiarization session, to measure the participant in the same position for all appointments. Moreover, to avoid any evasive movements, the participant’s trunk was fixed with two “seatbelt-like” straps and an additional fixation was wrapped around the hip. Operating a remote control, the participant was asked to move their ankle joint with an angular velocity of maximum 5°/s into the maximum possible dorsiflexion position. The maximum position was maintained for a second, and the ankle joint was then moved back into neutral position. This procedure was performed three times, with a 5 s break in between each attempt. The highest ROM value (i.e., the highest dorsiflexion ankle angle) reached was taken for further analysis.

### Passive resistive torque (PRT)

In the same position as during the ROM measurement, the ankle joint was moved passively between neutral position (90°) and the participant’s individual dorsiflexion ROM (PRT_max_) with an angular velocity of 5 s^−1^, to avoid any reflexive muscle activation (Kubo et al. 2002). Five cycles were conducted, and the lowest value of the maximum dorsiflexion of the last three cycles was taken for further analysis. In addition, PRT at a given angle (PRT_fixed_) was determined at the maximum angle achieved in both trials (pre and post). To monitor if the participant remained completely relaxed, the muscle activation level was measured with sEMG continuously during the test. In the case of an activation, the whole procedure was repeated.

### Shear wave elastography (SWE)

For the SWE testing, an ultrasound scanner (Aixplorer V12.3, Supersonic Imaging, Aix-en-Provence, France) was used. A linear transducer array (4–15 MHz, SuperLinear 15–4, Vermon, Tours, France) measured the muscle stiffness of the GM and GL in SWE mode (musculoskeletal preset, penetration mode, smoothing level 5, persistence off, scale 0–450 kPa). The participant was in the same position as described for the ROM measurements. The proceeding PRT measurement was used as conditioning for the SWE, to ensure the same condition at every appointment. SWE measurements were performed with a handheld technique (according to Lacourpaille et al. (2012)) in neutral ankle joint position (90°). We used a reusable foil, where the individual characteristics of the participant’s skin and the probe position were marked. A B-mode picture of the first measurement was also taken, to ensure reliable measurements (see Reiner et al. 2021). The shear modulus measurement position of both muscles (i.e., the GM and GL) was at the proximal third between the popliteal fossa (medially and laterally, respectively) and the calcaneus. Care was taken to avoid any deformation of body tissue due to pressure applied by the ultrasound probe during the measurement procedure (Kot et al. 2012). Aligning the probe in plane with the muscle fascicles, the region of interest (ROI) of the SWE was defined as the maximum possible size between, but not including, the aponeuroses. The participant’s passivity in the tested muscle tissue was checked visually with sEMG throughout the testing phase. For each muscle, three videos with a duration of 15 s each were recorded. For the further analysis, the mean of five consecutive frames with the lowest standard deviation within the ROI (averaged values) within one video was taken. The shear modulus of a muscle was then calculated as the mean between the two closest values of the three videos (Morales-Artacho et al. 2017).

### Maximum voluntary isometric contraction (MVIC) peak torque

The MVIC peak torque measurements of the plantar flexors were performed in the same position as all the other tests (prone position, ankle = 90°, proper fixation on hip and trunk). Each participant performed three MVICs of 5 s each, with a break of 1 min in between each attempt. The participant was verbally encouraged to push as hard as possible during the trial. The highest value was considered for further analysis. As with the previous measurements, the sEMG-signal was also recorded during the test.

### Surface electromyography (sEMG)

The sEMG (myon 320; myon AG, Zurich, Switzerland) was assessed during all the measurements (ROM, PRT, SWE, and MVIC), with a sample rate of 2000 Hz. The skin was prepared and the surface electrodes (Blue Sensor N, Ambu A/S, Ballerup, Denmark) were positioned on the GL in the proximal third of the muscle belly according to the “European Recommendations for surface Electromyography” (SENIAM) (Hermens et al. 1999). The inter-electrode distance was 2 cm. The raw sEMG values were monitored live during the ROM, PRT, and SWE measurements. If necessary, from the MVIC trials, the sEMG signals were high pass filtered (10 Hz, Butterworth) and the root mean square (RMS, 50 ms window) was calculated.

### Seven-week stretching and foam rolling intervention

**Fig. 2:**
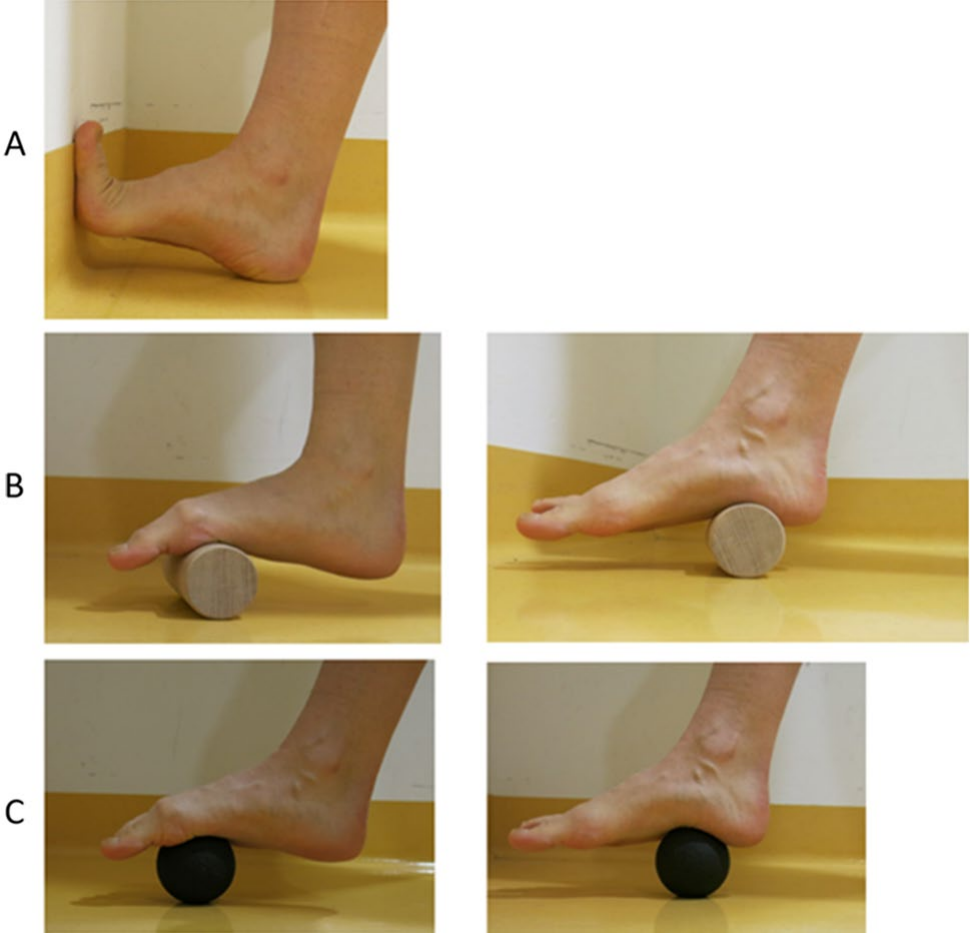
Figures of the stretching exercise (**A)** and foam rolling exercises (**B**, **C**) done within this study

Each participant was asked to perform three exercises for 5 min each (continuously, without a resting phase during each exercise), three times a week (recommendation: Monday, Wednesday, Friday), for a period of 7 weeks. Therefore, the total training duration per session was 15 min, with a total weekly training load of 45 min. Exercise 1 was a plantar foot sole stretch, where the participant positioned themselves in front of a wall, facing the wall. The participant’s toes and the ball of the foot were placed on the wall, while the heel stayed on the floor. To induce a stretch sensation, the ball of the foot should be pushed toward the floor. The participant was asked to maintain the position with the greatest tolerable stretching intensity. If the stretching intensity was too low in the standing position, the participant could either take a seat or cross their calf over the contralateral thigh and induce a plantar facia stretch by pulling their toes and the ball of their foot in the dorsal direction until they experienced a significant stretching intensity (see Fig. 2A). Exercise 2 was a rolling exercise performed with a custom-made wooden cylinder (diameter = 5.5 cm, length = 15 cm). The participant was asked to roll constantly along the plantar foot sole, linearly between the ball of the foot and the heel at a slow speed (i.e., ~ 2 s back and forth) and with as much pressure as tolerable (see Fig. 2B). Exercise 3 was another rolling exercise, but was performed with a foam roller ball (Ball 08, Blackroll, Bottighofen, CH). The participant was asked to roll constantly and slowly in small circles, and to cover the part of the sole of the foot between the ball of the foot and the heel (see Fig. 2C). The added pressure should again be as high as tolerable. Exercises 2 and 3 could be performed while sitting on a chair or standing with support while touching a wall or a desk. The participant was asked to perform the exercises in the same way during the whole intervention period. If interested, the participant was allowed to perform both exercises bilaterally.

The investigators supervised every single training session of the participants. In addition, the participants were asked to fill in a training diary. A minimum of 80% of the intervention volume was needed to positively finish the intervention.

### Control group

The participants in the control group were asked to perform a stretching program with three exercises of the pectoralis muscles, with the same instructions regarding duration, intensity, and period as the intervention group.

### Statistical analyses

Statistical analyses were performed using SPSS (Version 28, SPSS Inc., Chicago, Illinois). The tested variables were dorsiflexion ROM, PRT_max_, PRT_fixed_, MVIC plantar flexion peak torque, and shear modulus of the GM and GL. The baseline measurements did not differ between the two groups in any parameter. The data of all the parameters were tested for a normal distribution (Shapiro–Wilk test), and all were found to be normally distributed. A repeated measures ANOVA [factor: time (pre vs. post) and inter-subject factor: group (intervention vs. control)] was performed. If significant results were detected in the ANOVA, a paired *t* test was performed. Effect size partial eta square (η2) was defined as small, medium, and large for effect sizes greater than 0.01, 0.06, and 0.14, respectively (Cohen 1988). The alpha level was set to 0.05.

## Results

Significant time effects were revealed for PRT at maximum ROM (*p* < 0.01, F = 11.08; η2 = 0.28) and MVIC (*p* = 0.03, F = 5.13; η2 = 0.13) (Table 1). The increase for PRT at maximum ROM was 7.4 Nm in the intervention group and 3.6 Nm in the control group. Moreover, the increase in MVIC was 6.1 Nm, 2.2 Nm in the intervention group, control group, respectively.

**Table 1:** Pre- and post-values (mean ± standard deviation) and delta values, including the 95% confidence intervals (= CI) of the assessed parameters

	Plantar foot sole rolling and stretching group			Control group			Interaction effect		Time effect	
	PRE	POST	POST–PRE (CI 95%)	PRE	POST	POST–PRE (CI 95%)	*p* value	η^2^	*p* value	η^2^
ROM (°)	41.3 ± 7.9	43.3 ± 8.4	+ 2.0 (− 0.03 to 4.0)	41.6 ± 6.7	41.1 ± 7.6	− 0.5 (− 2.6 to 1.6)	0.08	0.09	0.28	0.03
PRT_max_ (Nm)	42.0 ± 16.8	49.4 ± 21.7	+ 7.4 (2.5 to 12.4)	48.6 ± 17.8	52.2 ± 17.1	+ 3.6 (− 1.4 to 8.6)	0.26	0.04	< 0.01	0.28
PRT_fixed_ (Nm)	41.0 ± 17.9	42.0 ± 19.4	+ 1.0 (− 1.7 to 3.8)	45.7 ± 18.3	46.5 ± 15.6	+ 0.8 (− 2.7 to 4.3)	0.91	0	0.39	0.03
Muscle stiffness GM (kPa)	9.4 ± 1.6	10.4 ± 3.6	+ 1.0 (− 0.5 to 2.6)	9.0 ± 1.9	8.9 ± 2.5	− 0.1 (− 1.3 to 1.0)	0.21	0.05	0.34	0.03
Muscle stiffness GL (kPa)	7.7 ± 1.7	7.7 ± 1.8	+ 0.04 (− 0.4 to 0.5)	7.2 ± 1.3	7.0 ± 1.9	− 0.2 (− 0.8 to 0.4)	0.52	0.01	0.67	0.01
MVIC (Nm)	136.5 ± 46.3	142.3 ± 48.3	+ 6.1 (− 0.3 to 12.4)	126.0 ± 26.6	128.1 ± 27.2	+ 2.2 (− 1.32 to 5.62)	0.29	0.03	0.03	0.13

Range of motion = ROM, passive resistive torque at max ankle angle ROM = PRT_max_, passive resistive torque at fixed ankle angle ROM = PRT_fixed_, gastrocnemius medialis = GM, gastrocnemius lateralis = GL, maximum voluntary isometric contraction = MVIC

There were no significant main effects for time with dorsiflexion ROM (*p* = 0.28; F = 1.19; η2 = 0.03), PRT at a fixed angle (*p* = 0.39; F = 0.76; η2 = 0.03), GM muscle stiffness (*p* = 0.34; F = 0.94; η2 = 0.03) or GL muscle stiffness (*p* = 0.67, F = 0.19; η2 = 0.01). Furthermore, there were no interaction effects for dorsiflexion ROM (*p* = 0.08; F = 3.26; η2 = 0.09), PRT at maximum ROM (*p* = 0.26, F = 1.32; η2 = 0.04), PRT at a fixed angle (*p* = 0.91; F = 0.01; η2 = 0.0), MVIC force (*p* = 0.29, F = 1.15; η2 = 0.03), GM muscle stiffness (*p* = 0.21; F = 1.67; η2 = 0.05) or GL muscle stiffness (*p* = 0.52, F = 0.42; η2 = 0.01) (Table 1).

## Discussion

The purpose of this study was to investigate the remote effects of a comprehensive 7-week combined stretching and FR training intervention of the plantar foot sole on the function (ROM, strength, passive torque) and structure (muscle stiffness) of the plantar flexors. The intervention did not lead to significantly different changes in any parameter, compared to a control group, but an increase of MVIC and PRT_max_ was seen in both groups. This indicates that a combined stretching and FR training for 7 weeks (3 × week for 15 min) of the plantar sole does not have a remote effect on the plantar flexors.

Dorsiflexion ROM of the ankle was unchanged following the 7-week intervention of combined stretching and FR training. However, there was a tendency of an interaction effect for dorsiflexion ROM (*p* = 0.08). The pre-to-post comparison showed an increase in ROM in the intervention group of 2.0° (*p* = 0.05), while the change in the control group was − 0.1° (*p* = 0.62). A potential increase in ROM in the intervention group was underlined by the 95% CI of − 0.03 to 4.0. However, compared to local interventions, the long-term stretching and FR of the foot sole seems to have no or only minor effects, as it has been found that direct stretching training of the lower leg for several weeks leads to increases in ankle joint ROM of > 5.0° in young healthy adults (e.g., Konrad and Tilp 2014; Medeiros and Martini 2018)).

To the best of the authors’ knowledge, this study is the first to have investigated the long-term remote effects of a plantar foot sole treatment via stretching and FR training on the dorsiflexion ankle ROM. However, previous studies have investigated the acute remote effects of FR along the superficial back line and reported increases in ROM in the hamstrings and lower back following a single FR treatment of the plantar foot sole (Grieve et al. 2015; Patel et al. 2016; Do et al. 2018). Similarly, Wilke et al. (2017) showed that stretching of the hamstrings also increases ROM in non-directly adjacent areas of the superficial back line, namely, the neck. A recent review reported that acute changes in non-local tissue ROM following a single bout of stretching are likely due to altered global pain perception (Behm et al. 2021). Although this is not yet clear, it can be assumed that an adjusted global change in pain perception following a single bout of FR or stretching will increase ROM in all non-treated joints, due to the conditioning effects.

Contrary to this theory, prior studies investigating myofascial connections have shown, for example, that ankle position and passive torque influence hip mobility, and vice versa (Palmer et al. 2015; Andrade et al. 2016; Marinho et al. 2017). Cadaveric studies have provided evidence for a potential transfer of tension between adjacent myofascial structures in the superficial back line (Krause et al. 2016); however, only a few in-vivo studies have suggested that force transmission along the superficial back line might be possible (Cruz-Montecinos et al. 2015; Wilke et al. 2020). Wilke et al. (2020), for example, investigated the superficial back line via SWE and found tissue displacement in the hamstrings region after passive ankle dorsiflexion. In addition, Ahmad et al. (2021) showed that hamstrings stretching in combination with suboccipital manual techniques is more effective in increasing hamstring muscles ROM than local stretching alone. In terms of force generation, it has been shown that ankle position and training of the foot muscles influence hamstrings strength (Sulowska et al. 2019; Nishida et al. 2022). These findings suggest strain transfer along the superficial back line via fascial pathways in the lower extremity and beyond. However, there is still a need for in-vivo studies regarding myofascial transmission along the superficial back line and other myofascial connections (Wilke et al. 2020).

With regard to long-term stretching and FR training studies on non-local ROM, in recent years, various training studies on the contralateral effects have been conducted. In these studies, unilateral interventions (i.e., FR, stretching) for several weeks were performed, and the ROM of the contralateral homologous muscle was obtained. Following both long-term FR (Kasahara et al. 2022) and stretching studies (Caldwell et al. 2019; Panidi et al. 2021; Nakamura et al. 2022), an increase in ROM has been reported in the contralateral limb. The potential mechanism for such an increase in ROM of the contralateral limb is related to increased pain perception.

In this study, a time effect was found in PRT_max_ (i.e., stretch tolerance), although it was indeed more pronounced and significant (post-hoc p = 0.01) in the intervention group (+ 7.4 ((95% CI 2.5–12.4)) than the control group (+ 3.6 ((95% CI − 1.4–8.6) *p* = 0.14). Even though a comprehensive stretching and FR intervention (45 min per week) of the plantar foot sole was used, no significant changes in muscle stiffness or PRT_fixed_ in the triceps surae were found in this study. This was likely because no mechanical load was applied on the triceps surae during the stretching and FR training. Consequently, this study confirmed that muscle stiffness of the gastrocnemii muscles and PRT_fixed_ are not affected by non-local and long-term stretching and FR training. These findings are underlined by a previous study which studied the contralateral effects of a high-intensity stretching training intervention (Nakamura et al. 2022) and FR training of the triceps surae muscles (Kasahara et al. 2022). Although the authors found an increase in dorsiflexion ankle ROM in the contralateral limb, no changes in muscle stiffness on the contralateral side occurred. Similar to the findings of the present study, these authors found increased stretch tolerance to be the main mechanism for an increase in ROM in the contralateral limb following a stretching (Nakamura et al. 2022) or FR training intervention (Kasahara et al. 2022) of several weeks. Thus, according to the findings of the previous studies on stretching and FR training on the contralateral effects and our current findings on the remote effects, it can be concluded that direct stress (e.g., stretching load) has to be applied on the respective tissue to cause changes in structure.

Another explanation for the unaltered muscle stiffness of the gastrocnemii in the current study might be the dense and strongly connected structures of the plantar foot sole (Chaudhry et al. 2008), which impede structural changes even when high loads are applied.

In the current study, plantar flexion MVIC was also assessed. The results showed a significant time effect, although the pairwise comparison and confidence intervals showed a more pronounced and significant (post-hoc p = 0.04) effect in the intervention group for an increase in MVIC (+ 4.5 (95% CI − 0.2–9.2)), compared to the control group (+ 4.0 (95% CI − 2.2–10.2) *p* = 0.18). With regard to the local effects of stretching, it has been reported that high-volume stretching for several weeks can induce an increase in muscle strength, which is likely due to the repeated tension applied on the target tissue (Yahata et al. 2021; Warneke et al. 2022a). With regard to long-term FR, a recent meta-analysis reported no changes in muscle performance following an FR training intervention (ES = 0.294; *p* = 0.281) (Konrad et al. 2022a). However, it has to be noted that the included studies in this meta-analysis did not perform a comprehensive FR training intervention, with the total load ranging from 1080 to 3685 s over the whole intervention duration. Thus, more load with repeated stretches during FR might be required to induce changes in muscle performance, as has been seen in stretching training (Yahata et al. 2021; Warneke et al. 2022a). The findings on the remote effects of the current study are in agreement with a study which investigated the contralateral effects of stretching and FR training on MVIC. Kasahara et al. (2022) reported a non-significant increase in the contralateral plantar flexion MVIC of 0.64% and 2.02% following a 6-week conventional FR and vibration FR training intervention of the ipsilateral limb, respectively. A potential mechanism for the increase in plantar flexion MVIC seen in the current study might be the activation of the lower limb muscles (i.e., tibialis anterior, triceps surae), to support balance during the repeated stretching and FR exercises. Kokkonen et al. (2007) suggested that the increase in muscle performance seen after 10 weeks of stretching might not be related to the stretching stimulus itself, but instead could be due to the stabilization tasks performed during stretching (e.g., standing on the contralateral leg while stretching the ipsilateral leg). Similarly, Zahiri et al. (2022) showed that the muscle activity of the core muscles during a plank or reverse plank position is similar to that with quadriceps and hamstrings FR, respectively. Hence, tasks other than the intervention itself might be responsible for the adaptations in MVIC seen in the current study. A further explanation for the increase in plantar flexion MVIC throughout the intervention period in the current study might be that the participants were recreational athletes, and hence were following individual training regimes. Consequently, this would also explain the significant time effect in MVIC, indicating an increase in both the intervention group and control group.

There are some limitations of the current study. First, the control group has performed stretching exercises of the upper limbs and hence, a potential cross-over training effect in ROM as has been seen after a single bout of stretching (Behm et al. 2021) or FR (Konrad et al. 2023) cannot be ruled out completely. Second, since the intervention was performed as a combination of stretching and FR exercises, an isolated effect of either stretching or FR cannot be assessed.

## Conclusions 

This study is the first to have investigated the remote effects of a 7-week combined stretching and FR training intervention of the plantar foot sole on the function and structure of the triceps surae. The results indicate no or only minor remote effects for this intervention. A non-significant increase in ROM in the intervention group might be explained by an increase in stretch tolerance (PRT_max_), but not by changes in the muscle structure (muscle stiffness), which was unaltered. Increases in plantar flexion MVIC were also observed in the control group, indicating that the 7-week intervention of the plantar foot sole was not the main reason for these changes.

## Data Availability

The original contributions presented in the study are included in the article. Further inquiries can be directed to the corresponding author.

## Abbreviations

CI: Confidence intervals
ES: Effect size
FR: Foam rolling
GM: Gastrocnemius medialis
GL: Gastrocnemius lateralis
MVIC: Maximum voluntary isometric contraction
PRT: Passive resistive torque
PRT_fixed_: Passive resistive torque at a given angle
PRT_max_: Passive resistive torque at participant’s individual dorsiflexion range of motion
ROM: Range of motion
sEMG: Surface electromyography
SWE: Shear wave elastography
